# Aichi Virus Shedding in High Concentrations in Patients with Acute Diarrhea

**DOI:** 10.3201/eid1708.101556

**Published:** 2011-08

**Authors:** Jan Felix Drexler, Sigrid Baumgarte, Luciano Kleber de Souza Luna, Monika Eschbach-Bludau, Alexander N. Lukashev, Christian Drosten

**Affiliations:** Author affiliations: University of Bonn Medical Centre, Bonn, Germany (J.F. Drexler, M. Eschbach-Bludau, C. Drosten);; Institute of Hygiene and the Environment, Hamburg, Germany (S. Baumgarte);; Bernhard Nocht Institute for Tropical Medicine, Hamburg (L.K. de Souza Luna);; Chumakov Institute of Poliomyelitis and Viral Encephalitides, Moscow, Russia (A.N. Lukashev)

**Keywords:** Picornaviridae infections, human aichi virus, Germany, communicable diseases, real time RT-PCR, virus shedding, viruses, dispatch

## Abstract

We assessed Aichi virus shedding in patients with gastroenteritis and negative test results for other viral and bacterial infections. High concentrations of up to 1.32 × 10^12^ RNA copies/g stool were found in 10 (2.0%) of 499 outpatients sampled in northern Germany, 2004. These data substantiate Aichi virus pathogenicity in humans.

The family *Picornaviridae* includes 12 established genera, and representatives of 5 of these have been found in humans (*Enterovirus, Hepatovirus, Parechovirus, Cardiovirus,* and *Kobuvirus*). Among those, human pathogenicity has been proven consistently only for enteroviruses (including polioviruses), hepatitis A virus, and parechoviruses. Several as-yet-unclassified picornaviruses have been found over the past few years in humans, termed cosavirus, klassevirus, and salivirus (*1**–**3*). For gastrointestinal pathogens, data on virus quantity in stool can exclude ingestion from nutritional sources of viruses that may be detected but do not replicate in the human gut. Prevalence studies with appropriate control groups and proof of the absence of co-infections with other pathogens are required to provide evidence in favor of human pathogenicity. For most of the novel viruses, these data are still awaited.

A novel human picornavirus termed Aichi virus (AiV; genus *Kobuvirus*), was described initially in 1991 (*4*) and epidemiologically linked with spontaneous and food-associated diarrhea in humans (*5**,**6*). Recently, it was also detected in sewage-polluted water (*7*). However, no quantitative data of AiV shedding have become available so far, possibly because of technical peculiarities such as high genomic GC content (≈60%) and strong RNA secondary structures, which may have contributed to a lack of sequence information and prevented more precise molecular detection. In this study, we analyzed well-established cohorts of patients with gastroenteritis and an appropriate control group. Stool samples from patients who had negative test results for other common viruses and bacterial infections showed high AiV shedding by highly sensitive real-time reverse transcription PCR (RT-PCR), thereby substantiating AiV human pathogenicity.

## The Study

The picornavirus 5′ untranslated region (5′ UTR) has proven the most suitable genomic target for molecular detection (*8**,**9*). At the time of this study, only 5 AiV complete genomes were available in GenBank, complicating selection of reliable oligonucleotides for universal detection. A nested RT-PCR encompassing the AiV 5′ UTR was developed (Table 1) and used for screening of stool samples collected in northern Germany from outpatients with gastroenteritis. The first subcohort of this collection consisted of 499 patients with gastroenteritis; samples were collected evenly from January through December 2004 in a prospective study on acute community-acquired diarrhea by 47 general practitioners in Bremen, northern Germany (*10*). The second subcohort consisted of 39 control patients without symptoms of gastroenteritis seen by the same physicians. The third subcohort consisted of 118 patients with diarrhea linked to outbreak scenarios involving canteen food (n = 36), kindergartens (n = 54), or retirement homes (n = 28).

**Table 1:** PCR oligonucleotides used for AiV amplification and quantification, Germany, 2004*

ID no.	Sequence, 5′ → 3′	Position†	Genome location	Orientation	RT-PCR type	Usage
AiV-F65	CACCGTTACTCCATTCAGCTTCTTC	65–89	5′ UTR	+	Nested, 1st round‡	Determination of suitable genomic target region for quantitative real-time RT-PCR
AiV-F69	GTTACTCCATTCAGCTTCTTCGGAAC	69–94	5′ UTR	+	Nested, 2nd round§	
AiV-R1039	CAGGATTGGACATCAGAATCATAGAG	1039–1064	Leader	–	Nested, 2nd round§	
AiV-R1049	GGATAGAACCAGGATTGGACATCAG	1049–1073	Leader	–	Nested, 1st round‡	
AiV-F274	CCAGCCTGACGTATCACAGG	274–293	5′ UTR	+	Real-time¶	Viral RNA quantification
AiV-R313	AAGCTGCTCACGTGGCAATTGTG	313–335	5′ UTR	–	Real-time¶	
AiV-P294	FAM-CTGTGTGAAGYCC-MGBNFQ	294–306	5′ UTR	+ (probe)	Real-time¶	
AiV-F2984	CAGGCATTCATCTCYGCAGGTGAA	2984–3007	VP1	+	Nested, 1st round‡	Determination of viral genotype
AiV-F2995	CTCYGCAGGTGAATCCTTCAACGT	2995–3018	VP1	+	Nested, 2nd round§	
AiV-R3881	GATGGCCCAGTGGACGTAGGT	3881–3901	VP1	–	Nested, 2nd round§	
AiV-R3884	TTGCGGATGGCCCAGTGGACGTA	3884–3906	VP1	–	Nested, 1st round‡	

*AiV, Aichi virus; ID, identification; RT-PCR, reverse transcription PCR; UTR, untranslated region. †Relative to AiV GenBank accession no. AB040749. ‡25-µL QIAGEN OneStep RT-PCR reactions as described by the manufacturer (QIAGEN, Hilden, Germany) used 400 nmol/L each of 1st-round primers, 1 µg bovine serum albumin, and 5 µL RNA extract. Amplification involved 30 min at 50°C; 15 min at 95°C; 10 cycles of 20 s at 94°C, 30 s starting at 60°C with a decrease of 1°C per cycle, and 50 s at 72°C; and 40 cycles of 20 s at 95°C, 30 s at 54°C, and 50 s at 72°C; and a final elongation step of 5 min at 72°C. §50-µL Platinum Taq reactions as described by the manufacturer (Invitrogen, Karlsruhe, Germany) used 1 µL of 1st-round PCR product, 2.5 mmol/L MgCl_2_, and 400 nmol/L each of 2nd-round primers. Amplification involved 3 min at 94°C and 45 cycles of 20 s at 94°C, 30 s at 60°C, and 40 s at 72°C. ¶25-µL QIAGEN OneStep RT-PCR reactions used 3 µL of RNA extract, 600 nmol/L of each primer, and 320 nmol/L of the probe. Cycling in an Applied Biosystems (Darmstadt, German) 7700 SDS instrument involved the following steps: 55°C for 15 min, 95°C for 15 min, and 45 cycles of 95°C for 15 s and 58°C for 30 s (fluorescence measured).

We purified viral RNA from stool samples by using the QIAGEN Viral RNA and DNA Stool Mini Kits (QIAGEN, Hilden, Germany) as described (*8*); 9 samples were positive for AiV by nested RT-PCR. The 5′ UTR sequences of these viruses were determined and deposited in GenBank (accession nos. GQ927704–GQ927712). With additionally available sequence data, real-time RT-PCR targeting conserved regions of the viral 5′ UTR was developed (Table 1). Assay sensitivity was determined to be ≈1.5 copies per reaction by using photometrically quantified in vitro cRNA transcripts, as described (*9*).

**Figure 1:**
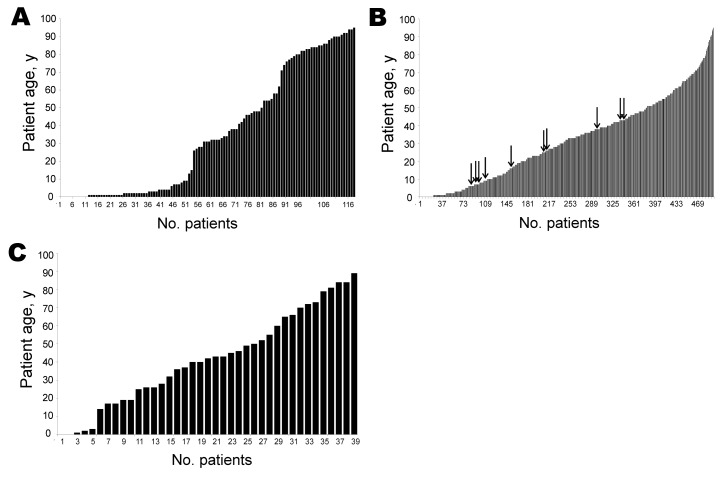
Age distribution of cohorts tested in study of Aichi virus in patients with acute diarrhea, Germany. A) Patients from food-associated diarrhea outbreaks (kindergartens, canteens, or retirement homes); B) outpatients seen for gastroenteritis by general practitioners; C) nongastroenteritis control patients for the outpatient study cohort. Arrows indicate patients who had positive test results for Aichi virus by real-time reverse transcription PCR.

Retesting of all samples with this highly sensitive assay increased the AiV detection rate, yielding 10 positive samples. All case-patients were part of the subcohort of symptomatic outpatients seen by general practitioners (Figure 1). In this cohort, AiV was detected at a 2.0% rate (10/499 patients). No patients from the control group (n = 39) or from foodborne outbreaks (n = 118) had positive test results for AiV. However, this difference was not statistically significant for any group comparison (Fisher exact test, 2-tailed p>0.05 for all). As shown in Table 2, all AiV-positive patients had abdominal pain, diarrhea, or nausea. No other virus commonly associated with diarrhea was detected in any patient, including norovirus, rotavirus, adenovirus, astrovirus, parechovirus, or enterovirus. A bacterial cause of disease was ruled out by using standard culture methods (*10*). No clear association with a foodborne etiology, sociologic risk factor (including travel history), or contact with animals was observed. As shown in Table 2, high RNA copy numbers of up to 1.32 × 10^12^ per gram of stool (mean 1.32 × 10^11^, median = 1.82 × 10^7^) were found in patients with positive test results.

**Table 2:** Characteristics of patients positive for Aichi virus, Germany, 2004*

Sample ID no.	Sampling date	Patient age, y/sex	Animal contact	Diarrhea	Emesis	Symptomatic contact person	Recent travel history	Other symptoms	Suspicious food ingested	Virus concentration†
D/VI 2169	Oct 19	7/M	No	Yes	Yes	Mother	No	Abdominal pain	None	1.30 × 10^12^
D/VI 2244	Nov 2	9/M	No	Yes	No	None	No	Abdominal pain	None	1.37 × 10^7^
D/VI 2287	Nov 8	27/M	No	Yes	No	None	No	None	None	6.77 × 10^7^
D/VI 2321	Nov 10	26/F	No	No	No	None	No	Abdominal pain	Fast-food chicken nuggets	6.09 × 10^9^
D/VI 2359	Nov 16	16/M	2 birds	Yes	No	None	No	Abdominal pain	Ground pork	2.79 × 10^6^
D/VI 2524	Dec 4	7/M	No	Yes	No	None	Italy	Abdominal pain	Chicken	4.21 × 10^7^
D/VI 2528	Dec 2	43/F	No	No	Yes	Daughter	No	Abdominal pain	None	4.42 × 10^6^
D/VI 2535	Dec 2	6/F	No	No	Yes	Mother	No	Throat pain	None	2.27 × 10^7^
D/VI 2582	Dec 6	38/F	Cat	Yes	Yes	None	No	Abdominal pain	Minced meat	9.99 × 10^6^
D/VI 2591	Dec 7	43/F	Dog	Yes	Yes	None	No	None	None	1.08 × 10^2^

*No patients had fever (>38.5°C) or co-infections. All samples were tested for norovirus, rotavirus, adenovirus, astrovirus, parechovirus, enterovirus, and common bacterial pathogens. ID, identification. †RNA copies/g stool.

**Figure 2:**
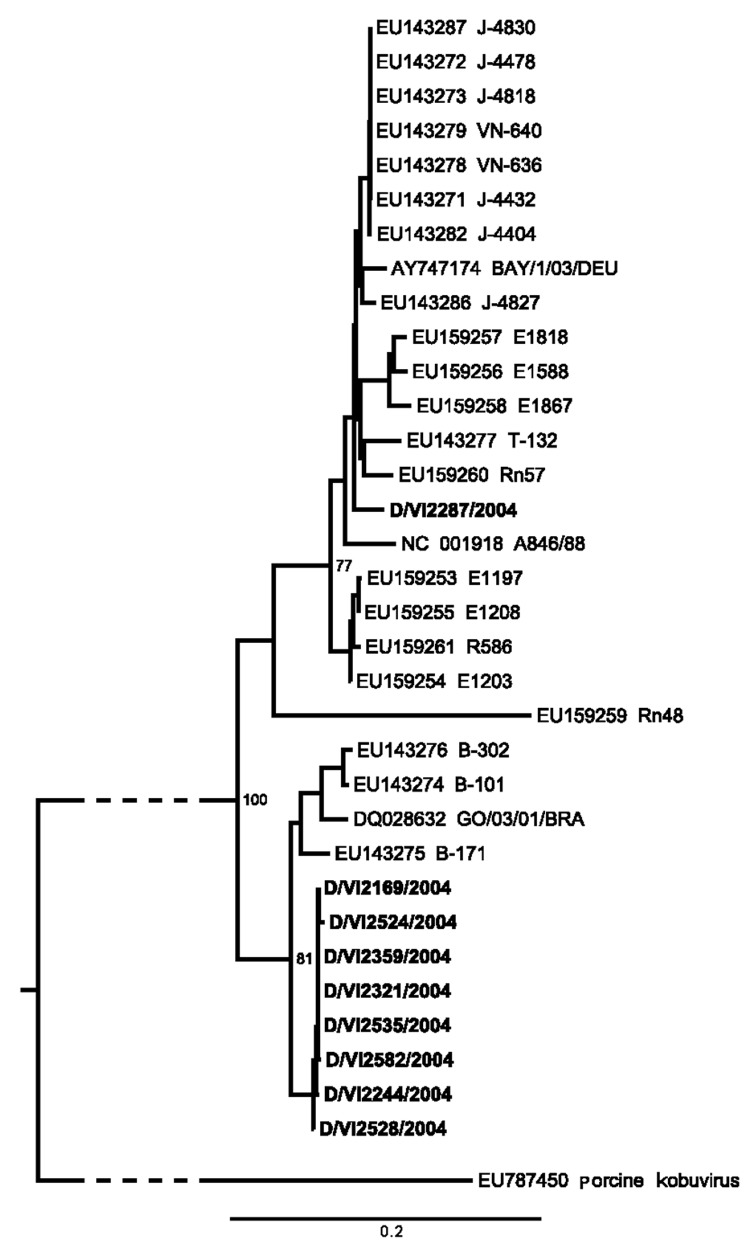
Neighbor-joining phylogeny of Aichi virus (AiV) viral protein 1 gene of strains from study of AiV in patients with acute diarrhea (**boldface**), Germany, compared with strains from GenBank. The tree was generated by using MEGA4 (www.megasoftware.net) using the maximum-composite likelihood nucleotide substitution model and complete deletion option. Porcine kobuvirus was used as an outgroup (branch truncated as indicated by slashed lines). Bootstrap values from 1,000 reiterations are depicted next to root points. The final dataset corresponded to nucleotide positions 3,034–3,663 in AiV GenBank accession no. AB040749. Scale bar indicates number of base substitutions per site.

Although samples had been collected throughout 2004, all AiV-positive cases occurred during 8 weeks from October to December and originated from a geographically restricted area within the city of Bremen. To verify if this temporal and geographic accumulation of cases represented a point-source outbreak, we amplified and sequenced the entire viral protein (VP) 1 gene, which is commonly used for picornavirus typing, from 9/10 samples (GenBank accession nos. GQ927704–GQ927712). Failure of VP1 amplification in sample D/VI2591 was probably caused by low virus concentration. As shown in Figure 2, a total of 8 samples formed a distinct phylogenetic cluster within AiV genotype B. The first 3 strains, sampled from October 19 through November 10 (Table 2), were almost identical in VP1, with only 1 strain (D/VI2244) diverging by 2 synonymous substitutions. All other samples (November 16–December 7) showed a VP1 nucleotide diversity of up to 0.8% (2–7/864 nt) and an amino acid diversity of up to 1.4% (1–4/288 residues) in comparison to the 3 initially sampled specimens and to each other. Strain D/VI2287, sampled November 8, belonged to AiV genotype A, with nucleotide and amino acid differences of up to 13.2% and 5.4% from the genotype B strains.

## Conclusions

For many of the recently described picornaviruses, human pathogenicity is still under study. With the advent of metagenomics, the description of multiple novel viruses can be expected. Although it is generally difficult to generate sufficiently large and appropriately sampled control groups in studies on respiratory and enteric diseases, quantitative data on virus shedding and proof of monocausality can contribute to confirm the link of novel viruses to human disease. For AiV, the high concentrations found in several samples in this study provide support for viral replication in humans. However, virus shedding appeared unrelated to the severity of symptoms, and clinical presentations were generally mild. Contrary to the findings of previous studies from France and Tunisia (*5**,**6*), no food association could be observed in this study. In agreement with some, but not all, published reports (*5**,**11*), no case of AiV-associated gastroenteritis from this study had apparent co-infections, further supporting AiV pathogenicity in humans. The overall 2.0% detection rate of AiV in stool samples from outpatients with gastroenteritis is compatible with detection rates in recent studies from several European and Asian countries (*5**,**12*). Surprisingly, AiV infection affected all age groups. This was in sharp contrast to parechoviruses and cardioviruses detected predominantly in patients <6 years of age in the same study cohorts (*8**,**9*) and indicated different modes of transmission and maintenance of these genetically related picornaviruses at the population level. Similarly, the lower AiV infection rate described here was consistent with the 51.0% seroprevalence rate described in German infants <2 years of age (*13*) compared with >75.0% described for cardioviruses and parechoviruses (*14**,**15*).

The geographic and temporal accumulation of cases, together with the observed sequence variation, supports locally and temporally restricted circulation of AiV with human-to-human transmission, rather than a point-source epidemic pattern as observed in foodborne infections. Fecal–oral human-to-human transmission would be facilitated by the high fecal virus concentrations in some patients. Our data indicate that AiV can be considered an authentic human pathogen that can be transmitted from human to human.
